# Impedimetric Nanobiosensor for the Detection of SARS-CoV-2 Antigens and Antibodies

**DOI:** 10.1021/acssensors.2c01686

**Published:** 2023-02-10

**Authors:** Diana Isabel Sandoval Bojórquez, Željko Janićijević, Brenda Palestina Romero, Eduardo Sergio Oliveros Mata, Markus Laube, Anja Feldmann, Alexandra Kegler, Laura Drewitz, Ciarán Fowley, Jens Pietzsch, Juergen Fassbender, Torsten Tonn, Michael Bachmann, Larysa Baraban

**Affiliations:** † Institute of Radiopharmaceutical Cancer Research, Helmholtz-Zentrum Dresden-Rossendorf e.V. (HZDR), 01328 Dresden, Germany; ‡ Institute of Ion Beam Physics and Materials Research, Helmholtz-Zentrum Dresden-Rossendorf e.V. (HZDR), 01328 Dresden, Germany; § School of Sciences, Faculty of Chemistry and Food Chemistry, Technische Universität Dresden, 01307 Dresden, Germany; ∥ Tumor Immunology, University Cancer Center (UCC), University Hospital Carl Gustav Carus Dresden, Technische Universität Dresden, 01307 Dresden, Germany; ⊥ National Center for Tumor Diseases (NCT), Dresden, Germany. Faculty of Medicine and University Hospital Carl Gustav Carus, Technische Universität Dresden, 01307 Dresden, Germany; # German Cancer Research Center (DKFZ), 69120 Heidelberg, Germany; ∇ German Cancer Consortium (DKTK), 01309 Dresden, Germany; @ Transfusion Medicine, Med. Faculty Carl-Gustav Carus, Technische Universität Dresden, 01307 Dresden, Germany; $ Institute for Transfusion Medicine Dresden, German Red Cross Blood Donation Service North-East, 01307 Dresden, Germany

**Keywords:** impedimetric sensing, point-of-care testing, SARS-CoV-2, gold nanowires, impedance model, reliability, electrochemical impedance spectroscopy (EIS), surface plasmon resonance (SPR)

## Abstract

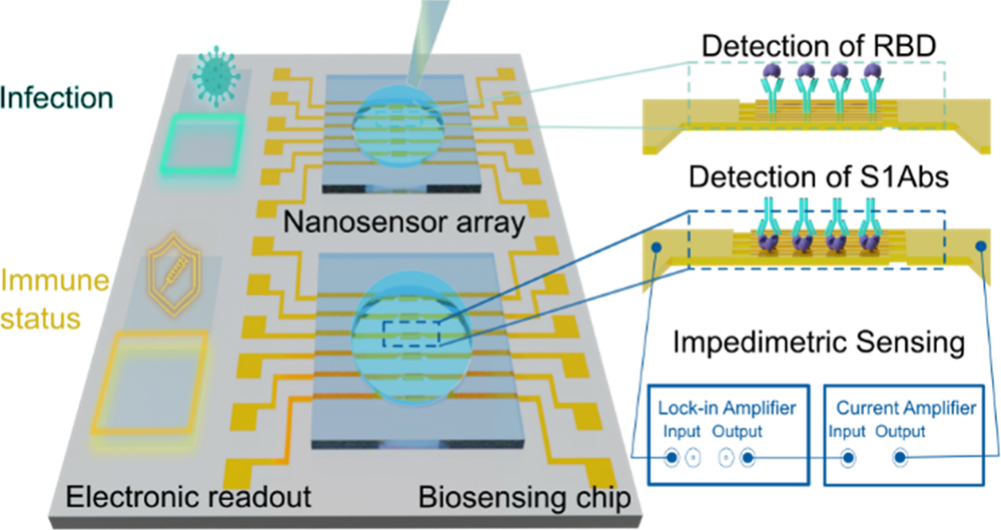

Detection of antigens and antibodies (Abs) is of great importance in determining the infection and immunity status of the population, as they are key parameters guiding the handling of pandemics. Current point-of-care (POC) devices are a convenient option for rapid screening; however, their sensitivity requires further improvement. We present an interdigitated gold nanowire-based impedance nanobiosensor to detect COVID-19-associated antigens (receptor-binding domain of S1 protein of the SARS-CoV-2 virus) and respective Abs appearing during and after infection. The electrochemical impedance spectroscopy technique was used to assess the changes in measured impedance resulting from the binding of respective analytes to the surface of the chip. After 20 min of incubation, the sensor devices demonstrate a high sensitivity of about 57 pS·s^*n*^ per concentration decade and a limit of detection (LOD) of 0.99 pg/mL for anti-SARS-CoV-2 Abs and a sensitivity of around 21 pS·s^*n*^ per concentration decade and an LOD of 0.14 pg/mL for the virus antigen detection. Finally, the analysis of clinical plasma samples demonstrates the applicability of the developed platform to assist clinicians and authorities in determining the infection or immunity status of the patients.

The COVID-19 pandemic has changed our society at all levels: from our personal life up to the agendas of national and global authorities that dedicate their efforts to containing the spreading of the disease and devising prevention strategies. These changes have come in the form of new scientific developments, healthcare measures, civil rights, and state-wide public health policies.^1^ Considering that our society is constantly evolving, the approach to tackle future pandemics will need continuous optimization. We have learned that to succeed in the eradication of new pandemics, we need to coordinate the efforts of doctors, pharmacists, scientists, engineers, politicians, citizens, and the media.

Nowadays, the world is searching for an adaptive solution to handle the negative impact of the pandemic on the economy, making fast decisions, and instigating effective sanitation measures, supported by quick testing systems.^2^ In this respect, the market for point-of-care (POC) devices has shown one of the highest dynamics during the COVID-19 pandemic, driven by offering innovative tools for making faster decisions. The POC device market is expected to grow at a compound annual growth rate of 9.4% from 2021 to 2028 considering the increment of chronic and infectious diseases as the driving factor.^3^ In this regard, the demand for the development of quality POC diagnostics will prevail with the pandemic.^3^

Studies have shown that POC lateral flow tests (LFTs) are an accurate alternative to reverse transcriptase-polymerase chain reaction (RT-PCR) as the gold standard technique for SARS-CoV-2 detection.^4,5^ The multiple benefits of using the LFT instead of RT-PCR include rapid detection, on-site testing, low cost, and operation without laboratory equipment.^4−6^ Apart from the systems that are already commercially available, there are multiple chip-based biosensor developments, based on polydimethylsiloxane (PDMS), papers, and other flexible materials, such as textiles, films, and carbon nanosheets.^7^ Although biosensors are a good alternative, there are remaining challenges that should be addressed. Namely, when compared to POC biosensors, quantitative RT-PCR shows much higher clinical sensitivity and specificity, which are 79 and 100%, respectively.^8^ On the other hand, the clinical sensitivity and specificity of the commercial POC biosensors [i.e., antibody (Ab) LFTs] are 86.43–93.75 and 90.63–100%, respectively.^7^ However, the main drawback of POC biosensors is that their sensitivity is dependent on the viral load. When the viral load is high, the sensitivity is 100%; in contrast, when the viral load is low, the sensitivity can drop below 10%.^5^ Viral load-dependent sensitivity may result in false-negative tests that affect adherence to quarantine and infection tracking measures. Thus, the biosensor community still must put efforts into addressing the aforementioned shortcomings, while also improving sensor performance stability and reliability. For the latter, additional control of the sensor stability and related output signal is necessary.

To improve the POC testing platforms, biosensor research is focused on the development of sensing systems that can be used for the detection of low viral loads and that can deliver fast and accurate results.^9−13^ One strategy to achieve this goal is the integration of nanostructures as sensing elements since the properties at the nanoscale offer attractive sensing performance.^14−17^ In particular, nanowires (NWs) have been used for the detection of different biomolecules, such as enzymes, proteins, and Abs, reaching a high sensitivity and low limit of detection (LOD).^12,18−21^ In combination with electrochemical impedance spectroscopy (EIS), metallic NWs can be used for label-free detection of antigen–Ab binding.^22−24^

EIS is a well-known measurement technique with many applications in different fields, such as the analysis of energy storage systems, assessment of material corrosion, and impedimetric sensing systems including biosensors.^25^ Impedimetric response, in general, carries information about different processes at the electrode/electrolyte interface including charge transfer, diffusive transport, and electrical double layer formation^26^ and properties of the measurement system such as solution resistance and electrode surface roughness^27^ or electrode porosity.^28^ Hence, EIS can relate the changes in electrical impedance to the modulation of physicochemical properties at the surface of the biosensing elements typically caused by the adhesion of analyte molecules.^29^ If the sensing area is functionalized with appropriate receptor molecules, the changes in impedance can be directly correlated with the binding of specific analytes. Compared to optical and electrochemical strategies, impedimetric sensing does not require the use of redox probes, has long-term stability, exhibits high levels of sensitivity, and offers the possibility of using portable and low-cost electronic interfaces.^10^

In this study, we address the aforementioned problems of POC biosensors focusing on the electronic sensors and implementing nanoscopic building blocks into the devices, to improve the sensitivity of the system. We developed interdigitated gold (Au) NWs as sensor elements to detect COVID-19-associated antigens and Abs appearing during and after infection with SARS-CoV-2. For this purpose, we used EIS to assess the changes produced by a binding event. The surface of the Au NWs was modified with appropriate receptor molecules to enable binding and detection of relevant COVID-19-related analytes. We thoroughly investigated the changes in the impedance profile, by accurately fitting the parameters of a lumped-element model representing the measurement system to the acquired impedance spectra. Finally, to demonstrate the applicability of the nanobiosensor, we performed the analysis of clinical plasma samples of seronegative and seropositive COVID-19 subjects. The promising features of our platform make it an attractive system for the evaluation of S1 Ab levels during the present pandemic and, prospectively, for the detection of other biomolecules.

## Materials and Methods

### Materials and Reagents

Poly(ethylene glycol)2-mercaptoethyl ether acetic acid (HS-PEG-COOH), *N*-Hydroxysuccinimide (NHS), ethanolamine, Tween 20, ammonium hydroxide (NH_4_OH), hydrogen peroxide (H_2_O_2_), hydrochloric acid (HCl), PBS tablets, and plain glass slides (25 mm × 75 mm × 1 mm) were purchased from Sigma-Aldrich. 1-Ethyl-3-(3-dimethylaminopropyl) carbodiimide (EDC) was obtained from Thermo Fischer Scientific Inc. HBS-P+ buffer and HEPES buffer pH 7 were purchased from Cytiva. SARS-CoV-2 Spike S1 (≥95%) protein receptor-binding domain (RBD) was provided by Trenzyme GmbH (>85%). Human anti-SARS-CoV-2 S1 monoclonal Ab (S1 Ab) was supplied by Creative Diagnostics. Polymethyl methacrylate (950 PMMA A6) positive electron beam resist, developer ma-D 533s, and negative photoresist ma-N 1420 were purchased from micro resist technology GmbH. Conductive resist (CR) AR-CP 5090.02 and adhesion promoter AR 300-80 were obtained from ALLRESIST GmbH. Acetone, isopropyl alcohol (IPA), ethanol, and isobutyl methylketone (MIBK) were supplied by Carl Roth GmbH. PDMS Sylgard 184 was purchased from Dow Corning. All materials were used as received with the analytic purification grade, without further modification or purification.

### Fabrication of the Sensing Platform

#### Fabrication of the External Contact Pads (ECPs)

Glass substrates (25 mm × 37 mm) were cleaned following RCA-1 (NH_4_OH/H_2_O_2_/H_2_O – 1:1:5) and RCA-2 (HCl/H_2_O_2_/H_2_O – 1:1:7) protocols. Then, the substrates were treated with O_2_ plasma (10 Pa, 15 sccm) at 400 W for 10 min. To increase the adhesion of the resist to the glass substrates, a layer of the adhesion promoter AR 300-80 was spin-coated over the substrates before spin-coating a 1.8 μm layer of ma-N 1420. For the patterning of the ECP, the substrates were exposed to UV light in a hard contact mode for 12 s using an MA6 mask aligner (SÜSS MicroTec Lithography GmbH) at 20 mW/cm^2^. To reveal the structures, the substrates were developed in maD533s for 2 min. Afterward, a thin film of Cr (10 nm)/Au (100 nm) was deposited using a CREAMET 600 e-beam evaporator (Creavac GmbH). Finally, the ECPs were formed after a metal lift-off process in acetone followed by a cleaning step with IPA.

#### Fabrication of the Interdigitated Au NWs

To obtain a highly hydrophilic surface, the substrates were treated with O_2_ plasma (10 Pa, 15 sccm) at 100 W for 1 min. Subsequently, a 330 nm layer of PMMA was spin-coated over the substrates followed by a 60 nm thick layer of CR. The interdigitated Au NWs were patterned by electron beam lithography (EBL) using an e-Line (Raith) EBL system at 150 μC/cm^2^. The substrates were developed in MIBK/IPA (1:3) for 1 min 45 s and washed with IPA. Afterward, a thin film of Cr (10 nm)/Au (50 nm) was deposited using a CREAMET 600 e-beam evaporator. Finally, the NWs were formed after the metal lift-off process in acetone followed by a cleaning step with IPA.

#### Fabrication of the PDMS Well

The fabrication of the PDMS well was achieved by drop-casting a 10:1 mixture of the polymer-curing agent over a 3″ silicon wafer. The mixture was then cured at 60 °C for 3 h. After this step, the PDMS block was removed from the wafer and cut into small pieces of 1.5 cm × 1.5 cm. A biopsy puncher of 0.5 cm in diameter was then used to make a hole in the middle of each piece. To form the wells, PDMS pieces were finally attached to the sensing chips using an uncured PDMS mixture as adhesive that was subsequently cured at 70 °C for 2 h. Before starting with the functionalization procedure, the surface of the NWs was cleaned with cyclic voltammetry (PalmSens4, PalmSens) from −0.5 to +0.5 V at a scan rate of 0.1 V/s for 10 cycles in PBS with 2 mM K_4_[Fe(CN)_6_]/K_3_[Fe(CN)_6_] (1:1), using a platinum wire as the reference electrode.

### Functionalization of the Sensor Surface and Analyte Sensing

The surface of the Au NWs was modified to attach the appropriate receptor molecule. The sensors were incubated with an HS-PEG_5k_-COOH solution in 10% v/v ethanol in water for 24 h (four consecutive incubations for 2 h with 10 nM solution, followed by a 16 h incubation with a 1 mM solution). To activate the −COOH groups, EDC/NHS (75–11.5 mg/mL) chemistry was employed. The sensors were incubated in this solution for 30 min. Then, the sensors were incubated with a solution containing the receptor molecule, either the RBD or the S1 Ab, at 20 μg/mL in HEPES buffer (pH 7) for 2 h. To block the remaining active sites, the sensors were incubated with a 1 mM ethanolamine solution at pH 8.5 for 1 h. The sensor was incubated with different concentrations of the analytes (from 1 fg/mL to 1 μg/mL in HBS-P+ buffer pH 7.4) for 20 min. After each incubation step, the sensors were washed three times with PBS buffer containing 0.1% Tween 20 (PBS-T). Afterward, the impedimetric measurements were performed in a PBS-T medium. The functionalization strategy was verified with surface plasmon resonance (SPR), where the immobilization buffer and the receptor molecule concentration were selected, and the activity and selectivity of the molecules were tested. The experimental details are fully described in the Supporting Information, section Surface Plasmon Resonance Analysis.

### Human Plasma Sample Procedures

The sensors were evaluated in human plasma samples from COVID-19-positive patients and a healthy subject. The sensor system has been tested with wild-type mutants, but it is adaptable to the multiple variants of the virus. Samples from 3 serology confirmed COVID-19 patients were obtained from the German Red Cross Dresden. A human plasma sample without neutralizing Abs against SARS-CoV-2 was provided by the University Hospital Carl Gustav Carus (negative control). Samples were stored at −20 °C until required for analysis. The concentration of Abs present in the clinical samples was determined by enzyme-linked immunosorbent assay (ELISA), and the details are fully described in the Supporting Information, section ELISA Assay. A simple sample dilution of 1:100 in PBS was required before testing the human plasma samples on the chip. The calibration curves were obtained by spiking S1 Abs (1 fg/mL–1 μg/mL) in the negative control sample (1:100 in PBS). The concentration of neutralizing Abs against SARS-CoV-2 in human plasma samples from three different patients was estimated by fitting the obtained data to their corresponding calibration curve using GraphPad Prism 9 (GraphPad Software, San Diego, California, USA). All subjects consented to participate in the study, which was approved by the local Ethics Committee.

### EIS Measurements and Data Analysis

All EIS measurements were performed with a lock-in amplifier (HF2LI, Zurich Instruments) coupled with a transimpedance current amplifier (HF2TA, Zurich Instruments). Excitation of the sensor was performed by applying a sinusoidal voltage of 1 V peak-to-peak amplitude in the frequency range from 100 Hz to 1 MHz. The voltage output of the transimpedance current amplifier was recorded in triplicate using the high-precision sweeper mode. Recorded data were utilized to calculate the impedance spectra that were subjected to further analysis and fitting based on a lumped-element model. Impedance spectra were validated using the Python-based analysis package impedance.py.^30^ Preprocessing, analysis, and fitting of the impedance spectra were carried out using a set of custom MATLAB scripts (MATLAB R2018b, The MathWorks, Inc., Natick, MA, USA). Noise measurements were performed in the high-precision sweeper mode by recording the standard deviation of the voltage signal at the input of the lock-in amplifier under the same experimental conditions used to obtain the impedance spectra. Acquired data were then used to calculate the noise amplitude spectral density and signal-to-noise ratio (SNR) of the sensor. Further details about the EIS and noise measurements, including the corresponding data analysis can be found in the Supporting Information (refer to sections Impedance Data Validation and Model Fitting Approach and Noise Measurements).

## Results and Discussion

### Fabrication and Operation Principle of the Impedimetric Nanobiosensor

**Figure 1:**
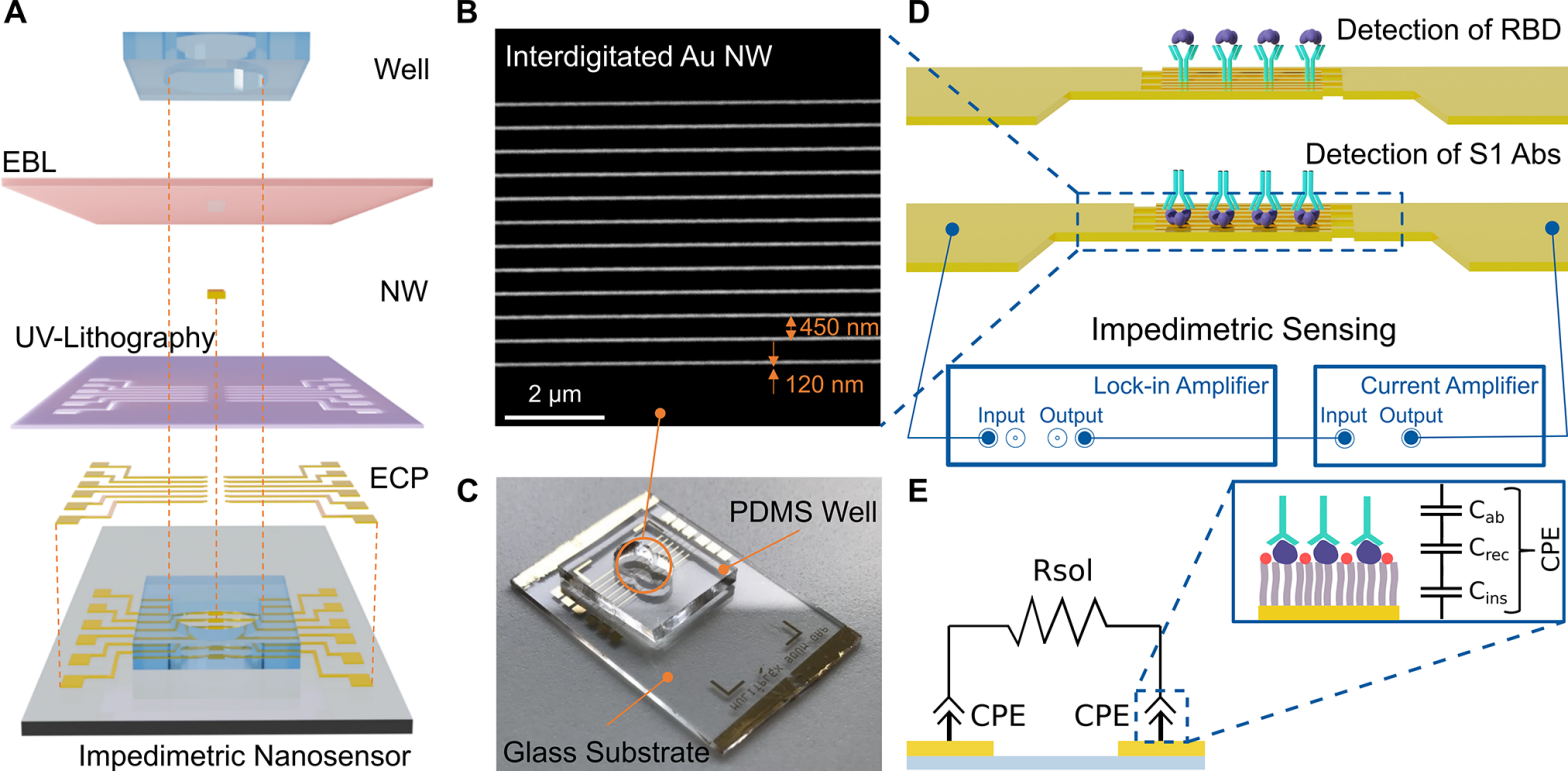
(A) Schematic representation of the impedimetric nanobiosensor fabrication and assembly: deposition of external contact pads (ECP) patterned by UV lithography, Au NWs patterned by EBL, and a PDMS well. (B) SEM image of the interdigitated Au NWs with an average width of 120 nm, length of 49 μm, and spacing of 450 nm. (C) Image of the complete sensor including a PDMS well of 100 μL volume for drop testing. The liquid sample of interest is dispensed in the well and allowed to incubate. (D) Schematic representation of the measurement setup. Functionalized Au NWs can detect the binding of either antigens or Abs by measuring the changes in the impedance using a lock-in amplifier coupled with a current amplifier. (E) Electrical equivalent circuit of the impedance model with the illustration of constant phase element (CPE) interpretation. CPE is a simplified circuit element describing the equivalent contribution of different molecular layers attached on top of the Au NW surface.

The use of diagnostic POC tests, which can detect infectious agents accurately and rapidly, is of great relevance at the moment. Furthermore, the possibility to detect several analytes, e.g., antigens and the corresponding neutralizing Abs would be an interesting solution, as worldwide we still suffer from a high COVID-19 infection rate, while we also try to determine the duration of immunity after infection and vaccination.^31−34^ In this work, we developed a nanoscopic biosensor chip consisting of six pairs of interdigitated Au NW devices for the detection of SARS-CoV-2 antigens and Abs. Devices were fabricated on glass slides by combining EBL for the fabrication of Au NWs with the standard UV lithography for producing the microscopic contact pads (see Figure 1A). The Au NWs were designed to have a width of 120 nm, length of 49 μm, and interelectrode spacing of 450 nm, as shown in Figure 1B. Finally, the measurement chamber (with a well diameter of ca. 1 cm and volume of ca. 100 μL) was fabricated by attaching a cured PDMS well to the glass. The PDMS well worked as a droplet container for the functionalization and testing procedures. Figure 1C shows the photograph of the complete nanobiosensor with designated main parts (design is described in our previous work^35^).

For the EIS measurements, the sensing chip terminals were connected to the measurement setup comprising a lock-in amplifier coupled to a current amplifier (Figure 1D). The lock-in amplifier was used to apply an AC input voltage excitation signal (1 V peak-to-peak sine wave) over a range of frequencies (100 Hz–1 MHz), and then record the changes in the output amplitude and phase of the measured voltage signal induced by analyte sensing. These variations were generated by the attachment of molecules to the surface of the Au NWs.

The response of the sensors can be analyzed in terms of an equivalent electrical circuit comprising lumped elements. Each circuit element models a specific contribution to the sensing behavior and can be related to the physical sensor components (including the NWs, medium, and molecules). Hence, impedance changes (calculated from the amplitude and phase variations of the measured voltage signal) can be correlated with the binding of molecules to the surface of Au NWs. Figure 1E shows the schematic illustration of the equivalent circuit representing the impedance model of the sensing system.

The equivalent electrical circuit of our impedance model comprises a resistor and two CPEs connected in series; its equivalent impedance *Z*_e_ can be calculated as follows:^26^
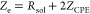
1where *R*_sol_ is the bulk resistance of electrolyte solution in the droplet and *Z*_CPE_ is the impedance of the electrical double layer interface between the Au NWs and analyte solution, in this case, represented by the CPE. The imperfect capacitive behavior of the double layer at the electrode surface is further described as
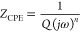
2where *Q* numerically corresponds to the admittance at angular frequency ω = 1 rad/s and *n* is the numeric exponent in the range from 0 to 1 describing the phase angle displacement compared to the ideal capacitor.

The employed impedance model is characteristic for impedimetric biosensing in non-Faradaic mode, using functionalized solid electrodes.^36^ Specific binding of the analyte to the biorecognition layer coating the surface of the solid electrode causes modulation of interfacial capacitance. When a binding event occurs, biomolecules displace the aqueous electrolyte or cause a transition to a different conformational state leading to an effective change in the capacitance of the biorecognition layer.^37,38^ A change in capacitance caused by analyte binding is then determined from the measured impedance spectra. Such a change in capacitance could be correlated with the values of CPE parameters *Q* and *n*. Apart from being useful in the characterization of the sensing response, values of *Q* and *n* can also serve as helpful indicators of impedance profile quality or sensing surface alterations caused by, e.g., output signal drift, failed surface functionalization, or sensor damage during operation.

We estimated the parameters of lumped elements in the equivalent circuit of the impedance model using a customized high-resolution fitting approach, coupled with initial validation of calculated impedance spectra (Figure S1). In further analysis, we exploit the robust impedance modeling and detailed characterization of the impedimetric response to assess the sensing sensitivity, quality, and reliability of the measurements of COVID-19-associated antigens and Abs.

### Functionalization of the Biosensor

**Figure 2:**
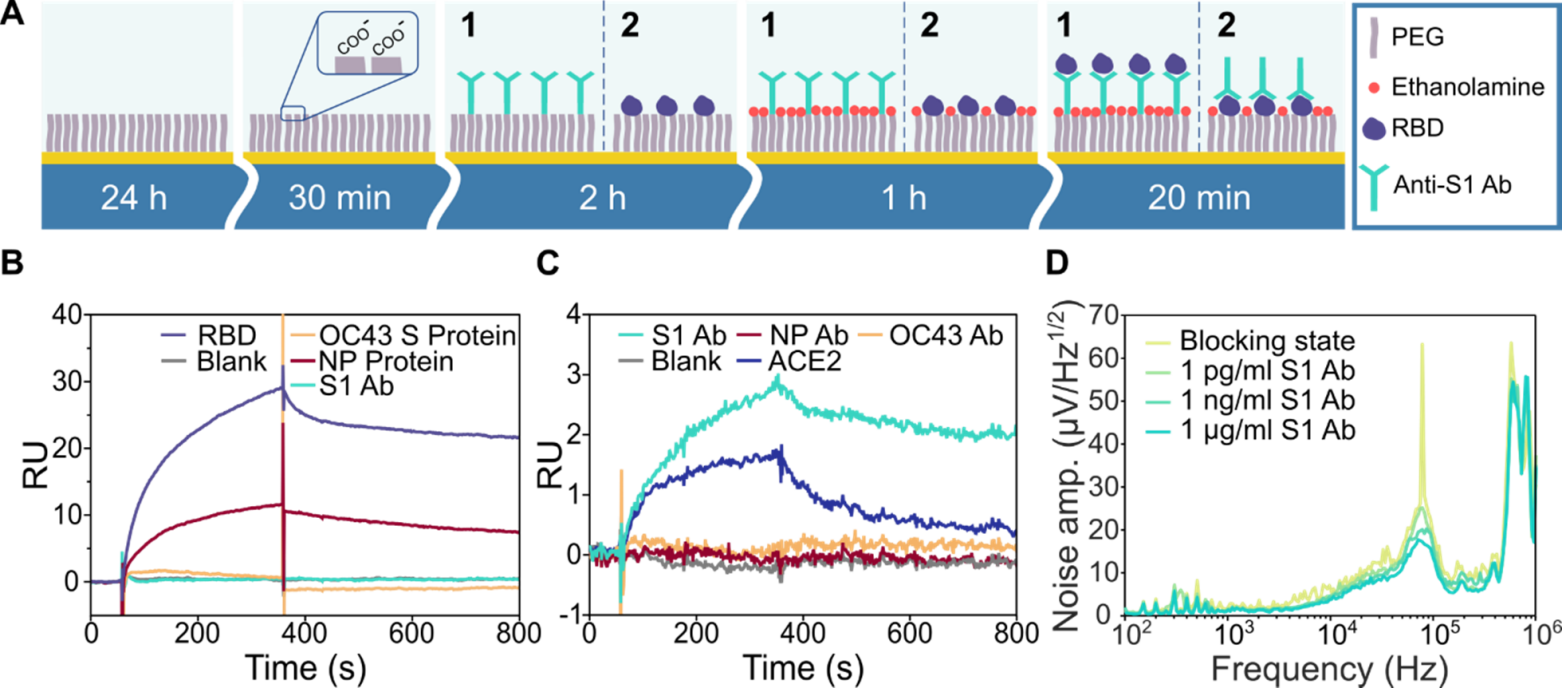
Functionalization of the nanobiosensor surface: (A) protocol of surface functionalization for the detection of 1-RBD and 2-S1 Abs, starting with a SAM formation followed by amine coupling of the captured molecules and the surface blocking with ethanolamine. SPR characterization of the surface functionalization used in (A) for (B) RBD and (C) S1 Ab as ligands. Change in the response units (RU) indicates binding of analytes to the surface and hence binding events. (D) Monitoring of S1 Ab analyte binding to the Au NW surface using noise amplitude spectral density.

For the specific detection of antigens and Abs present during and after contact with the SARS-CoV-2 virus, the surfaces of the Au NWs were functionalized with the corresponding receptor molecules. Figure 2A shows a schematic representation of the functionalization steps employed for the detection of the SARS-CoV-2 S1 protein RBD (route 1) and anti-SARS-CoV-2 IgG Abs (S1 Abs, route 2). First, the sensors were incubated with an HS-PEG_5k_-COOH solution to allow for uniform and saturated binding of the PEG via its thiol group to the Au surface. To activate the carboxylic acid groups for subsequent coupling steps to antigens or Abs, EDC/NHS chemistry was employed. Following the NW functionalization protocol 1 with S1 Abs, the platform can detect RBD molecules in a label-free form which serves as an indicator of infection with SARS-CoV-2. Vice versa, the functionalization with RBD via route 2 enables the detection of S1 Abs, which is relevant for immunity prevalence monitoring.

To validate the functionalization strategies for the detection of both analytes, each binding step was systematically verified using the gold standard SPR technique (Figure 2B,C). According to the NW surface modification, the plain Au surface of the SPR sensor chip was functionalized with HS-PEG_5k_-COOH solution to achieve the formation of the PEG layer followed by coupling to the S1 Ab (route 1, Figure 2B) and RBD (route 2, Figure 2C) via EDC/NHS chemistry. The SPR analysis indicated completion of PEG-layer formation within 24 h as visible from minor response changes at the end, which served as a reference for the NW modification described above (Figure S2). After coupling the ligands to the surface, an active and selective layer was obtained. For instance, the changes in the RU in the sensorgram shown in Figure 2C indicate that the RBD can bind to the S1 Abs and its natural binding partner human ACE2, while the negative controls anti-SARS-CoV-2 nucleocapsid protein antibody (NP Ab) and the anti-human coronavirus OC43 antibody (OC43 Ab) produce no signal, as expected. In this case, notably, ACE2 binding to RBD resulted in a markedly lower response than expected from the immobilization rate and a 1:1 binding kinetics. This might indicate that the more unselective EDC/NHS coupling via the amine groups of RBD principally is not optimal for this analyte ligand pair; however, other coupling or capturing strategies are typically available to enhance the activity of the ligand on the surface in future applications. On the other hand, the sensorgram in Figure 2B shows that the S1 Ab binds the RBD, while the negative control human coronavirus OC43 S protein and the S1 Ab itself produce no signal. Interestingly, the SARS-CoV-2 nucleocapsid protein also binds to the S1 Ab but this result would not affect the performance of the sensor since both antigens belong to the same virus and binding still results in a truly positive outcome. Hence, both coupling strategies based on a PEG-modified Au surface result in a biosensor surface suitable for SARS-CoV-2 Ab or antigen detection. Further details of the SPR experiments are given in the Supporting Information, section Surface Plasmon Resonance Analysis.

Using the NW-based nanobiosensor functionalized with RBD (route 2), the detection of the S1 Abs was also achieved by directly recording the spectral density of noise amplitude (Figure 2D). The calculated noise amplitude spectral density data show a clear trend of noise level reduction with the increase in S1 Ab concentration in the frequency range from 40 to 150 kHz, which is most apparent at the peak centered around 80 kHz (Figure 2D). This noise peak corresponds to the capacitive coupling of electrical disturbances in the surrounding medium by the Au NWs. Additional binding events enhance the surface coverage of Au NWs by forming the dielectric coating layer. Therefore, the passivation and electrical insulation of Au NWs improve noise performance and lead to decreased noise levels. A more detailed analysis including other noise profile aspects involved in impedance sensing is provided in the Supporting Information (see the section Noise Profile Analysis for Impedance Sensing and Figures S3, S4).

### Detection of Antigens and Abs from SARS-CoV-2

The main measurable parameter in our impedimetric nanobiosensor is the change in impedance compared to the blocking state, which is given by the following expression:
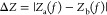
3where *Z*_a_(*f*) is the measured value of impedance modulus for the given concentration of the detected analyte at defined frequency *f* within the impedance spectrum, and *Z*_b_(*f*) is the measured value of impedance modulus at the same frequency in the blocking state.

**Figure 3:**
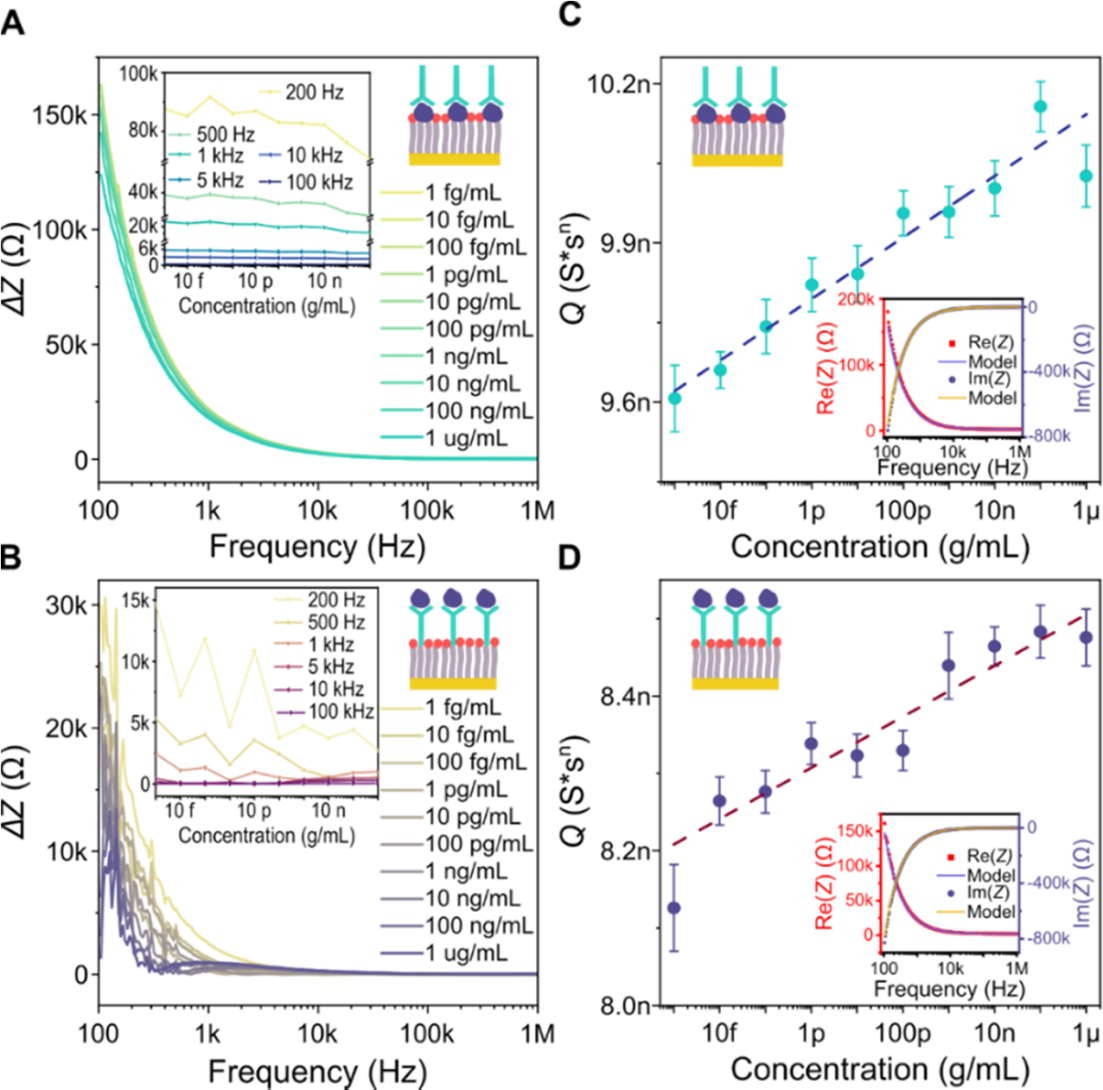
Dependence of change in impedance modulus (Δ*Z*) on frequency for different analyte concentrations of (A) S1 Ab and (B) S1 receptor-binding domain (RBD). Insets in (A) and (B) illustrate trends of Δ*Z* at specific frequencies with respect to the analyte concentration. Dependence of impedance model parameter *Q* on analyte concentration for (C) S1 Abs and (D) RBD. Insets in (C) and (D) show examples of impedance model-based fits used to derive the values of *Q* from real and imaginary parts of impedance spectra. All experimental measurements of analytes were performed in phosphate-buffered saline containing 0.1% Tween 20 (PBS-T) at ambient temperature. Error bars indicate the standard deviation for each measurement point (*N* = 3).

Representative frequency profiles of Δ*Z* values versus frequency were recorded using different concentrations of S1 Ab and RBD as analytes, respectively (Figure 3A,B), and the concentration dependency of Δ*Z* profiles obtained at distinct representative frequencies was extracted (corresponding insets in Figure 3A,B). The frequency dependence of Δ*Z* exhibits a general tendency of shifting toward lower Δ*Z* values with the increase of analyte concentration at low frequencies (approximately below 10 kHz), while such a tendency is diminished at higher frequencies. Obtained values of Δ*Z* are overall significantly higher for S1 Ab as the analyte, compared to RBD at the equivalent concentrations in the low-frequency range. The tendency of Δ*Z* change is inconsistent and noisier at very low frequencies (typically below 500 Hz), which is particularly visible in Figure 3B for the RBD analyte. The inconsistency is presumably correlated to the lower SNR, which is characteristic for this frequency range in our impedimetric measurement system (see Figure S4 in the Supporting Information). The described properties of the Δ*Z* profile in the frequency domain coincide with the expected capacitance change as the main driving effect of the sensor response to analyte binding. This effect should be also reflected in the change of frequency-independent parameter *Q* obtained when accurate fitting of the system is performed using the appropriate impedance model (for details refer to eqs 1 and 2 and Figure S5). Based on the impedance model fitting results for parameter *Q* (see Figure 3C,D), we can estimate the LOD (0.99 pg/mL for S1 Ab and 0.14 pg/mL for RBD) and sensitivity (57.13 ± 5.05 pS·s^*n*^ for S1 Ab and 20.83 ± 4.19 pS·s^*n*^ for RBD per concentration decade) for both analytes.

Figure 3C,D illustrates the trends of parameter *Q* change with analyte concentration for S1 Ab and RBD detection, respectively. The insets of Figure 3C,D show the representative fits obtained using the impedance model in both cases. The values of parameter *Q* increase with the analyte concentration in both cases, while they are higher overall and more consistent for S1 Ab detection when compared to RBD detection. Although highly accurate fitting can be obtained using the impedance model for both analytes, error margins of the measurement and deviations from the general trend limit the possibility for precise quantitative detection of analytes using Δ*Z* or *Q*.

### Analysis of Clinical Samples

**Figure 4:**
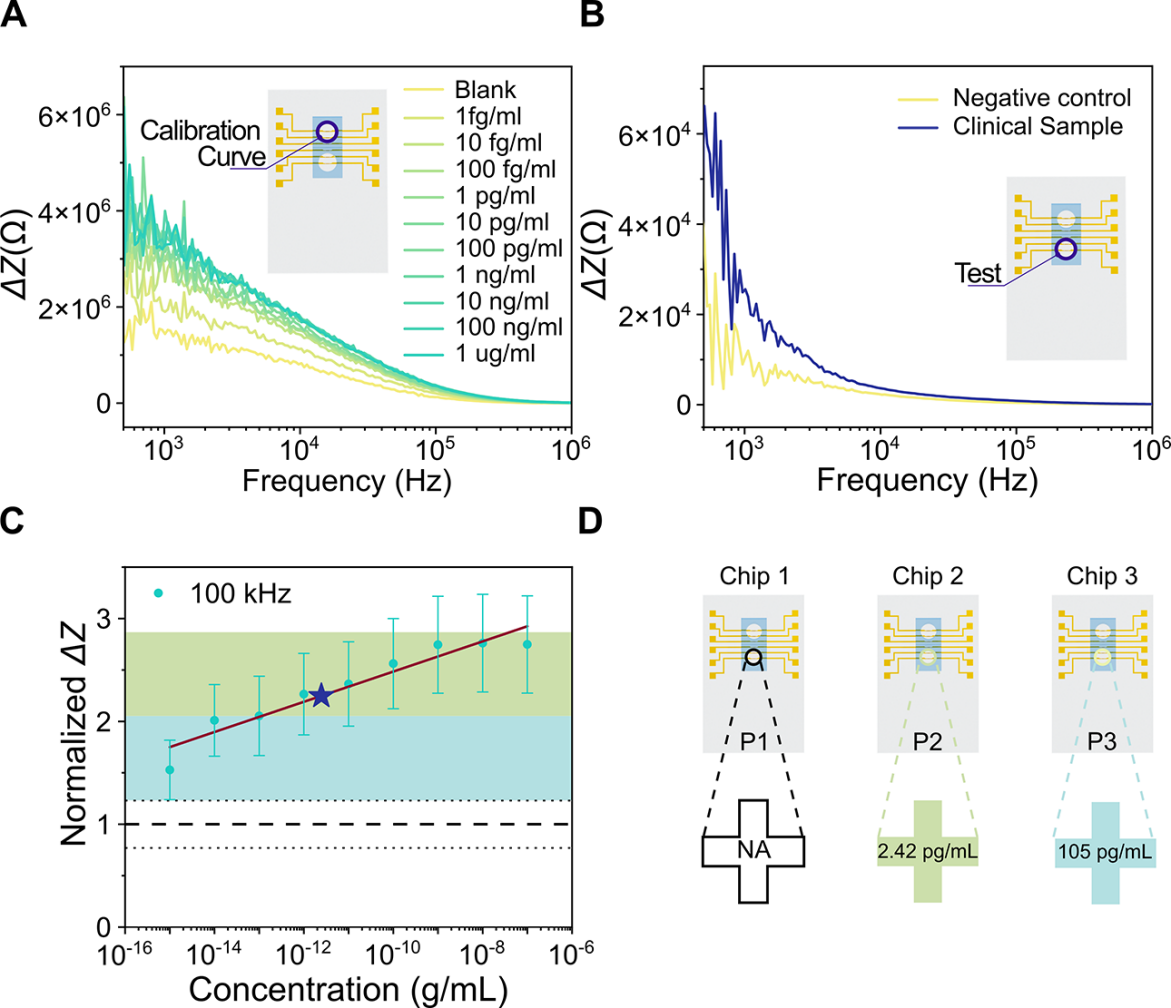
(A) Relative impedimetric sensor response from 1 fg/mL to 1 μg/mL of S1 Abs spiked in human plasma. The inset shows the layout of the chip indicating that the experiment was performed in the upper well. (B) Demonstration of the signal response of a seronegative (negative control) and a seropositive COVID-19 subject. The inset shows the layout of the chip indicating that the experiment was performed in the lower well of the same chip used in (A). (C) Calibration curve at 100 kHz extracted from (A). Values were normalized with respect to the blank. Dashed and dotted lines show the value of the blank and the relative standard deviation (see the section Experiments with Clinical Samples in the Supporting Information), respectively. Star represents the estimated value of the concentration of S1 Abs of the subject shown in (B), calculated from the fitted line. Colored regions represent reliability levels (see the section Experiments with Clinical Samples in the Supporting Information), where green indicates higher reliability than blue. (D) Comparison of the responses of three different chips for testing clinical samples from patients P1–P3. The crosses indicate that the samples tested were classified as positive for COVID-19 Abs. The color of the cross indicates the reliability level.

Clinical samples from seronegative and seropositive COVID-19 subjects were analyzed using ELISA and classified into three groups: (1) control (below 2 μg/mL); (2) moderate S1 Ab concentrations (2–10 μg/mL); and (3) high S1 Ab concentrations (above 10 μg/mL) (see Table S1). Details on ELISA experiments are given in the Supporting Information, section ELISA Assay. To verify the ability of our impedimetric nanobiosensor to assist in the analysis of the clinical samples, the PDMS reservoir was modified to have two separate sensing areas in the same chip as it can be seen in the insets of Figure 4A,B. The upper well was used to obtain a calibration curve (Figure 4A), while the lower well was used to test the Ab levels in the clinical samples (Figure 4B). The Au NWs in both wells were functionalized as described above (see Figure 2A and related text).

To obtain the calibration curve, diluted plasma (1:100 in PBS) from a seronegative subject was spiked with S1 Abs in the concentration range from 1 fg/mL to 1 μg/mL (the blank refers to the diluted plasma without S1 Abs). By using this approach, the Abs spiked in human plasma for the calibration curve were exposed to a similar environment to the naturally appearing Abs present in the human plasma samples after the infection with SARS-CoV-2. In Figure 4A, we can observe that with increasing the S1 Ab concentration, the change in impedance magnitude Δ*Z* increases. This is an opposite trend compared to the trend observed when using a physiological buffer (see Figure 3A). Trend reversal might result from the interaction of other elements present in complex biological fluids with the sensor surface.^39^ Such interaction can have a passivation effect that can influence the electrical performance of the nanobiosensor.^40,41^ Additionally, we can observe an increase in the noise on the curve presented in Figure 4A compared to Figure 3A. This suggests that the medium in which the molecules are suspended influences the noise.

In terms of the detection ability of the nanobiosensor, in Figure 4B, it is possible to observe a clear increase in the impedance change of the response when the clinical sample was tested compared to the negative control (1:100 diluted plasma in PBS from a seronegative subject). By extracting the values of impedance change at 100 kHz for both, the calibration well and the test well, and normalizing them with respect to the change produced by the blank (negative control), it was possible to construct a calibration curve and estimate the concentrations of the clinical sample and the negative control using interpolation (see Figure 4C and the Supporting Information, section Experiments with Clinical Samples). The colored regions shown in Figure 4C (and Figure S6) represent reliability levels (see the section Experiments with Clinical Samples in the Supporting Information), where the green region indicates higher reliability than the blue region. The interpolated value for the clinical sample shown in Figure 4C is well differentiated from the blank level and its estimated value (2.42 pg/mL) lies within the green region, which indicates that the result has high reliability.

Furthermore, two different chips were used to assess two additional clinical samples. Figure 4D shows a comparison of the chip responses when testing three different clinical samples. The three samples tested were classified as positive with different reliability levels. From the estimated values, it was possible to identify the presence of Abs in the samples from patients P2 and P3 within the green and blue regions, respectively. For patient P1, it was not possible to estimate the value using the calibration curve of chip 1 due to improper sensor performance (see Figure S6). However, by comparing the response of the negative control with the response from patient sample P1 (see Figure S7), it was possible to observe a clear increase in the impedance change when the clinical sample was tested, giving a qualitative positive result.

### Evaluation of the Sensor Performance

The reproducibility of electrochemical nanobiosensors is a common issue in laboratory-scale or batch fabrication.^42^ Although technical limitations can arise during various stages of the fabrication process at the nanoscale (in our case of Au NWs on the glass substrate), the key challenges of impedimetric biosensor performance typically relate to the quality and dynamics of the complex sensing interface.^43^ In the case of the non-Faradaic impedimetric approach, the high quality of the first insulating layer determining the Au electrode surface coverage is crucial for good sensitivity and reproducibility of the biosensor.^38^

As demonstrated above, our sensors show consistent properties of the impedance profile when operating properly in a physiological buffer. However, their sensing performance should also be evaluated to assess interchip and intrachip variability. While the batch-to-batch variation in the properties of our impedimetric biosensor is still relatively high due to the laboratory fabrication conditions, the intrachip variability is promising. We focus here on the intrachip analysis of sensor reproducibility and the interpretation of performance variation using the impedance model (for details about the impedance model refer to the section Fabrication and Operation Principle of the Impedimetric Nanobiosensor and the section Impedance Data Validation and Model Fitting Approach in the Supporting Information).

**Figure 5:**
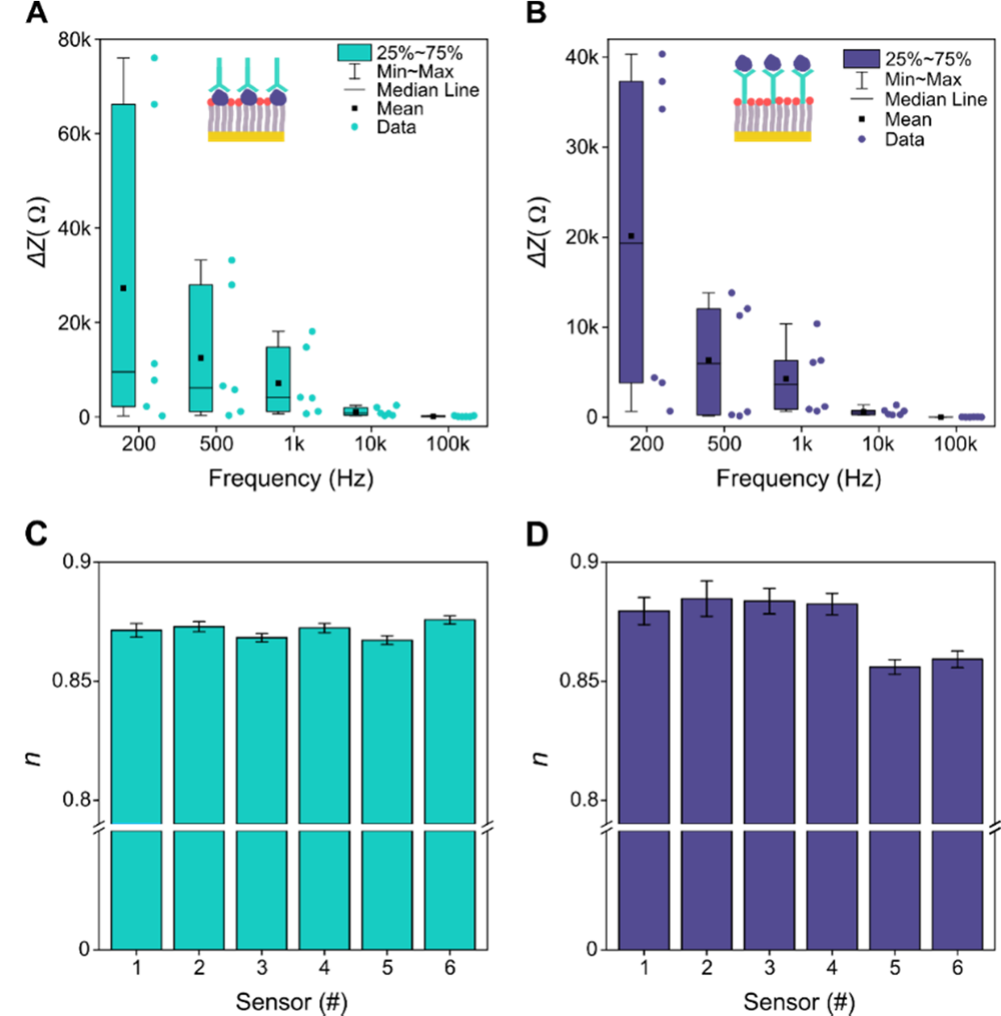
Analysis of the intra-chip reproducibility of impedimetric sensing performance. Box-and-whisker plots of Δ*Z* at specific frequencies for six sensors in the chip measured during the detection of 100 ng/mL of (A) S1 Ab and (B) RBD in PBS-T medium at ambient temperature. Values of numeric exponent *n* extracted from fitting the impedance model to the corresponding profiles of impedance response for (C) S1 Ab and (D) RBD detection at a concentration of 100 ng/mL in the PBS-T medium at ambient temperature. Error bars indicate the standard deviation for each measurement point (*N* = 3). Reproducibility of sensor response can be correlated with the precise matching of *n* between individual sensors.

The intrachip reproducibility was studied by testing six sensors for the detection of both S1 Ab and RBD at a fixed concentration of 100 ng/mL. Figure 5A,B shows the box-and-whisker plots of Δ*Z* at relevant frequencies for detecting S1 Ab and RBD, respectively. Measured values of Δ*Z* exhibit clustering behavior indicating a similar response of the sensors within the chip. Sensors with highly similar responses are adjacent to each other on the chip and share almost equivalent values of parameter *n* extracted from the impedance model and illustrated in Figure 5C,D for the detection of S1 Ab and RBD, respectively. *n* remains practically constant for the same sensor even for significantly different concentrations of the measured analytes allowing for relevant comparison in terms of sensor performance. The value of *n* can be a good indicator of electrode surface coverage quality as values of *n* closer to 1 typically reflect a smoother surface and the more stable capacitive response of impedimetric biosensing in the non-Faradaic regime as reported previously in the study by Castiello et al.^27^ Even a relatively small variation in the value of *n* can be related to significant changes in the impedimetric response of our sensors. The values of *n* in our sensors are lower than 0.9, indicating lower surface coverage quality compared to the microelectrode-based impedimetric sensor studied by Castiello et al.^27^ Such a result can be attributed to the more heterogeneous surface properties of the fabricated Au NWs, including greater variations in surface roughness and deviations in Au NW width arising as inherent consequences of the fabrication process.

## Conclusions

We have developed and fully characterized the non-Faradaic impedimetric nanobiosensor based on interdigitated Au NWs to detect both COVID-19-associated antigens (RBD domains of SARS-CoV-2 virus) and respective Abs appearing during and after infection with the virus. The EIS technique was used to assess the changes in measured impedance produced by the binding of the respective analyte to the surface of the chip. After 20 min of incubation, sensor devices demonstrated high sensitivity of about 57 pS·s^*n*^ per concentration decade and an LOD of 0.99 pg/mL for anti-SARS-CoV-2 Ab detection, while the sensitivity of around 21 pS·s^*n*^ per concentration decade and LOD of 0.14 pg/mL corresponded to the virus antigen detection. We hypothesize that these excellent values are achieved due to the miniaturization of the sensors and the implementation of the nanoscopic interdigitating NWs into the device. Further, the analysis of the clinical plasma samples demonstrated the applicability of the developed platform for determining the immunity status of the patients and thereby assisting the decision making of clinicians and authorities.

By using the nanobiosensor, it was possible to determine the presence of Abs in human plasma samples. However, to achieve a fully quantitative assessment of the samples, the SNR of the sensing system should be increased. Nevertheless, the layout of the nanobiosensor allowed us to perform an on-chip calibration that enables the determination of reliability levels. The dynamic range of the sensor hinders the possibility of measuring whole plasma samples directly, and yet a simple sample dilution in the buffer is sufficient to reach the appropriate range.

The continuous development of electronic biosensor technologies will open the possibility to have price-competitive mass production of biosensing systems, in turn also reducing the chip-to-chip variability encountered during laboratory-scale fabrication. In addition, the fabrication of sensors including nanostructures can unlock the integration potential by significantly decreasing the sensing areas and thereby allowing the sensing of multiple molecules within a single chip.

Finally, since a full screening in a wide range of frequencies is only needed during the research and development stage, we believe that impedimetric sensors based on interdigitated NW elements could be implemented to record the impedimetric response only at selected frequencies of interest. This simplification will offer the possibility to create miniaturized and reliable readout platforms based on established electronics design to screen diverse biomarkers and pathogens in the future with a POC device.

## Supporting Information Available

Additional experimental and methodological details regarding surface plasmon resonance analysis, ELISA assay, impedance data validation and model fitting, noise analysis, and clinical sample analysis including representative results (PDF)

## ABBREVIATIONS

Abs: antibodies
Au NWs: gold nanowires
ECP: external contact pads
EBL: electron beam lithography
EIS: electrochemical impedance spectroscopy
ELISA: enzyme-linked immunosorbent assay
LOD: limit of detection
LFT: lateral flow test
PDMS: polydimethylsiloxane
POC: point-of-care
RBD: receptor-binding domain
RT-PCR: real-time polymerase chain reaction
SNR: signal-to-noise-ratio
SPR: surface plasmon resonance.
