# Correction to Impedimetric Nanobiosensor for the Detection of SARS-CoV-2 Antigens and Antibodies

**DOI:** 10.1021/acssensors.3c01503

**Published:** 2023-08-24

**Authors:** Diana Isabel Sandoval Bojórquez, Željko Janićijević, Brenda Palestina Romero, Eduardo Sergio Oliveros Mata, Markus Laube, Anja Feldmann, Alexandra Kegler, Laura Drewitz, Ciarán Fowley, Jens Pietzsch, Juergen Fassbender, Torsten Tonn, Michael Bachmann, Larysa Baraban

The original acknowledgment is being changed to that given below.

All coauthors approve this addition and correction to the article.

