# Supplementary material for: Impedimetric Nanobiosensor for the Detection of SARS-CoV-2 Antigens and Antibodies
Source: ACS Sens. 2023 Feb 10;8(2):576–86. doi: 10.1021/acssensors.2c01686 (PMC9940615; doi:10.1021/acssensors.2c01686)
Supplement: Supplementary file 1 — se2c01686_si_001.pdf [file se2c01686_si_001.pdf]

Supporting Information

Impedimetric nanobiosensor for the detection of SARS-CoV-2 antigens and antibodies

Diana I. Sandoval Bojórquez[1§], Željko Janićijević[1§], Brenda Palestina Romero[1], Eduardo S. Oliveros Mata[2], Markus Laube[1], Anja Feldmann[1], Alexandra Kegler[1], Laura Drewitz[1], Ciarán Fowley[2], Jens Pietzsch[1,3], Juergen Fassbender[2], Torsten Tonn[8], Michael Bachmann[1,4-7*], Larysa Baraban[1*]

[1]Institute of Radiopharmaceutical Cancer Research, Helmholtz-Zentrum Dresden-Rossendorf e. V. (HZDR), 01328 Dresden, Germany

[2]Institute of Ion Beam Physics and Materials Research, Helmholtz-Zentrum Dresden-Rossendorf e.V. (HZDR), 01328 Dresden, Germany

[3]Technische Universität Dresden, School of Sciences, Faculty of Chemistry and Food Chemistry, 01307 Dresden, Germany

[4]Tumor Immunology, University Cancer Center (UCC), University Hospital Carl Gustav Carus Dresden, Technische Universität Dresden, 01307 Dresden, Germany

[5]National Center for Tumor Diseases (NCT), Dresden, Germany. Faculty of Medicine and University Hospital Carl Gustav Carus, Technische Universität Dresden, 01307 Dresden, Germany.

[6]German Cancer Research Center (DKFZ), Heidelberg, Germany

[7]German Cancer Consortium (DKTK), Dresden, Germany

[8]Department for Transfusion Medicine, German Red Cross Blood Donation Service North-East, Dresden, Germany

*Corresponding authors: Larysa Baraban, l.baraban@hzdr.de; Michael Bachmann, m.bachmann@hzdr.de

[§]These authors made equal contributions to the manuscript: Diana I. Sandoval Bojórquez, Željko Janićijević

Surface plasmon resonance analysis

The surface plasmon resonance (SPR) analyses were performed on a Biacore T200 (GE Healthcare). Experiments were performed using the 'SIA kit Au' (Cytiva Lifescience, Part-No.: BR100405) starting from the plain gold sensor chips. The sensors chips were cleaned before use by short immersion in ethanol (HPLC grade) and drying under a stream of argon followed by mounting of the sensor on the holder according to the manufacturer's instructions. After inserting the sensor chip into the system and equilibration using water as eluent, all flow cells (FCs) were normalized resulting in RU of 29700-31300 in water. Functionalization of the gold surface was performed by sequential injection of a solution of 5 mg/mL HS-PEG_{5k}-COOH (Sigma Aldrich, CAS 165729-81-7) in 10% v/v ethanol in water at a flow rate of 2 µL/min. Incubation of flow cells 1 and 2 or 3 and 4 was performed 12 times for 2 h with an intermediate pause of 120 s to evaluate the response after each 2 h injection. The final response difference resulting from surface modification was found to be between 4200-6050 RU.

Experiments with RBD on the active surface: HBS-P+ buffer (Cytiva) was used for further SPR experiments. SARS-CoV-2 S1 receptor binding domain (RBD, His-tagged recombinant human protein, Trenzyme GmbH, 27.5 kDa), was immobilized on FC2 or FC4 of the sensor chip using the amine coupling kit (GE Healthcare) and HBS-P+ (Cytiva) as running buffer. The amine coupling procedure comprises the surface activation by EDC/NHS, the coupling step using a solution of RBD at a concentration of 20 µg/mL in 10 mM HEPES (pH = 7.0) for 900 s, and the blocking of the surface using 1 M ethanolamine (pH 8.5). Using this coupling procedure, 230.6 ± 14.7 (n = 3) RU of RBD was immobilized on the sensor surface. A reference cell was prepared by blank immobilization (FC1 or FC3). Surface binding tests were performed with the following analytes: hACE-2 (Trenzyme GmbH, tag-free, 80 kDa, 60 nM in HBS-P+), mAb S1 (Creative Diagnostics, tag-free, 150 kDa, 2 nM in HBS-P+), mAb NP (Creative Diagnostics, tag-free, 900 kDa, 2 nM in HBS-P+), mAb human OC43 (CUSABIO Technology LLC, tag-free, 150 kDa, 2 nM in HBS-P+).

Experiments with mAb AntiS1 on the active surface: HBS-P+ buffer (Cytiva) was used for further SPR experiments. mAb S1 (Creative Diagnostics, tag-free, 150 kDa) was immobilized on FC4 of the sensor chip using the amine coupling kit (GE Healthcare) and HBS-P+ (Cytiva) as running buffer. The amine coupling procedure comprises the surface activation by EDC/NHS, the coupling step using a solution of mAb S1 at a concentration of 20 µg/mL in 10 mM HEPES (pH = 7.0) for 900 s, and the blocking of the surface using 1 M ethanolamine (pH 8.5). Using this coupling procedure, 154 RU (n = 1) of mAb S1 was immobilized on the sensor surface. A reference cell was prepared by blank immobilization (FC3). Surface binding tests were performed with the following analytes: RBD (His-tagged recombinant human protein, Trenzyme, 27.5 kDa, 60 nM in HBS-P+), SARS CoV-2 NP protein (Creative Diagnostics, His tagged, 47 kDa, 60 nM in HBS-P+), S Protein OC43 (Creative Diagnostics, His tagged, 145.1 kDa, 60 nM in HBS-P+), mAb anti-S1 (Creative Diagnostics, tag-free, 150 kDa, 60 nM in HBS-P+).

Binding tests: The analytes as described above were injected over the two flow cells (FC1 and 2 or FC3 and 4) at a flow rate of 30 µL/min and at a temperature of 25 °C. The association and dissociation time was 300 s and 480 s, respectively. The surfaces were regenerated by a

sequence of glycine treatment (10 mM, pH = 1.5, 30 s) followed by a stabilization period of 500 s. Three start-up cycles and one analysis cycle using only HBS-P+ buffer as ‘analyte’ were performed at the beginning of the experiments. Data were collected at a rate of 10 Hz and subsequently analyzed using the Biacore Evaluation software 3.0. Sensorgrams were reference subtracted (FC2-1 or FC4-3) and Y-adjusted to the baseline before analyte injection.

ELISA assay

All clinical samples were analyzed using enzyme-linked immunosorbent assay (ELISA) to determine the concentration of anti-SARS-CoV-2 antibodies.

ELISA tests were performed in a 96-well plate (Corning 96 Well EIA/RIA Assay Microplate). Measurements on clinical samples, as well as positive and negative controls, were performed in triplicate. After each incubation step, the wells were washed 3 times with washing buffer (PBS-T). The wells were coated with 100 µL of a 2 µg/mL solution of the SARS-CoV-2 S1 protein receptor-binding domain (RBD) suspended in ELISA coating buffer (OptEIA kit, BD) and incubated at 4 °C overnight. To avoid unspecific absorption, the remaining active sites were blocked with a blocking solution (PBS-T + 3% w/w skim milk powder) for 1 h at room temperature. After blocking, the wells were incubated with 7 standards to obtain the calibration curve (from 1.562 ng/mL to 100 ng/mL), 3 samples from COVID-19 recovered patients (1:1000 v/v dilution) and 1 sample from an unexposed subject (1:100 v/v dilution). Additional wells incubated with anti-SARS-CoV-2 nucleocapsid protein antibody and anti-hCoV OC43 S protein antibody at 1 µg/mL served as negative controls. The sample dilutions were made in PBS-T + 1% w/w skim milk powder. Subsequently, HRP-conjugated secondary antibody (1:1000 v/v in PBS-T) was added to each well and incubated for 1 h at ambient temperature. TMB substrate solution (OptEIA kit, BD) was then added to each well and allowed to react for 10 min. The reaction was stopped by adding a stop solution (OptEIA kit, BD). Finally, the optical density was measured at 450 nm with a GloMax Explorer (Promega, Germany) plate reader.

Data analysis was performed with GraphPad Prism 9 (GraphPad Software, San Diego, CA, USA). The calibration curve was fitted using a symmetric sigmoidal four-parameter logistic equation. To calculate the concentrations from the ELISA test results, the data points were interpolated from the standard curve.

Impedance data validation and model fitting approach

We evaluated the validity of calculated impedance spectra to confirm the electrically passive nature of the impedimetric sensor during the measurements. For the validation of impedance spectra, we utilized the Python package impedance.py[1] intended for the in-depth analysis of data obtained by electrochemical impedance spectroscopy. The protocol we used for data validation within the package impedance.py is based on the linear Kramers-Kronig (lin-KK) method of validity testing proposed by Boukamp[2] and later improved by Schönleber et al[3]. In brief, this method fits the impedance spectra using a model comprising the series connection of a resistor and multiple RC elements defined by logarithmically distributed time constants within the frequency range of acquired impedance data. The model is as follows:

$$Z_{\mathrm{KK}}(\omega) = R_0 + \sum_{k=1}^{\mathrm{N}} \frac{R_k}{1 + j\omega\tau_k} \tag{S.1}$$

where ω is the angular frequency, $Z_{\mathrm{KK}}(\omega)$ is the impedance model function defined by the lin-KK method, R_0 and R_k are variable fitting parameters, N is the number of RC elements, and τ_k are predefined values of RC element time constants distributed logarithmically between the minimum and maximum angular frequencies of the impedance spectrum. Further details can be found in Schönleber et al.[3] and impedance.py package documentation.

We performed non-linear regression to fit the impedance spectra by minimizing the total weighted sum of squared errors (WSSE) comprising the contributions of SSEs obtained by fitting the real and imaginary part of impedance data independently using the impedance model. For this purpose, we employed a simplified algorithm based on the gradient descent approach where individual parameters of the impedance model (R_{sol}, Q, and n) were varied in fixed small steps individually during each iteration. Small iterative changes in parameter values were approved and their direction (increase or decrease) reversed in such a manner to continuously reduce total WSSE until its value is stabilized and convergence achieved. To minimize the biasing of parameter estimates, we employed the function-proportional weighting approach[4] to calculate the WSSE as follows:

$$\mathrm{WSSE} = \sum_{i=1}^{\mathrm{L}} \left(\frac{Z_{\mathrm{XM}}(i) - Z_{\mathrm{XS}}(i)}{Z_{\mathrm{XS}}(i)}\right)^2 + \left(\frac{Z_{\mathrm{YM}}(i) - Z_{\mathrm{YS}}(i)}{Z_{\mathrm{YS}}(i)}\right)^2 \tag{S.2}$$

where L designates the number of logarithmically distributed frequency points in the measured impedance spectrum, $Z_{\mathrm{XM}}(i)$ designates the real part of the measured impedance at frequency point i, $Z_{\mathrm{XS}}(i)$ designates the real part of the simulated impedance at frequency point i, $Z_{\mathrm{YM}}(i)$ designates the imaginary part of the measured impedance at frequency point i, and $Z_{\mathrm{YS}}(i)$ designates the imaginary part of the simulated impedance at frequency point i.

To estimate fit quality, we calculated the residual standard errors $\mathrm{RSE_X}$ and $\mathrm{RSE_Y}$ corresponding to the fitting of real and imaginary parts of impedance data, respectively, using the expressions:

$$\mathrm{RSE_X} = \sqrt{\frac{1}{\mathrm{L}-3}\sum_{i=1}^{\mathrm{L}}\left(Z_{\mathrm{XM}}(i) - Z_{\mathrm{XS}}(i)\right)^2} \tag{S.3}$$

$$\mathrm{RSE_Y} = \sqrt{\frac{1}{\mathrm{L}-3}\sum_{i=1}^{\mathrm{L}}\left(Z_{\mathrm{YM}}(i) - Z_{\mathrm{YS}}(i)\right)^2} \tag{S.4}$$

where L-3 is the number of degrees of freedom due to the existence of three fitting parameters.

Initial values of fitting parameters were chosen based on the acquired impedance data and expected impedimetric sensor properties. Initial numerical values for R_{sol} equaled the values of the real part of impedance at the highest frequency for each impedance spectrum. The initial numerical values for Q were chosen between 10^{-9} and 10^{-8}, and for n between 0.8 and 1.

In the first fitting stage, all parameters were iteratively modified to estimate their values and variation within different impedance spectra recorded during a series of sensing measurements with the same gold NW pair. During calibration, R_{sol} and n showed very stable values and relative random deviation smaller than 0.5%. In this case, we introduced the second fitting stage where R_{sol} and n were fixed to their average values for a single gold NW pair. In this stage, only Q was slowly varied to determine the impedimetric response more reliably and reduce the execution time of the algorithm.

Noise measurements

Noise measurements were conducted using the lock-in amplifier (HF2LI, Zurich Instruments) in the frequency range from 100 Hz to 1 MHz with the same excitation and signal coupling parameters as during the impedance measurements of sensing performance. The lock-in amplifier was adjusted to measure the standard deviation of the input voltage signal in high precision sweeper mode. The standard deviation was calculated based on at least 1000 samples acquired per one of 200 logarithmically distributed frequency points within the range. The filter of 4th order was employed for the acquisition with maximum frequency bandwidth set to 1.25 MHz without overlap and bandwidth suppression set to 60 dB. Data acquisition per frequency point was performed for at least 100 ms or 50 time constants (whichever is longer) using the settling time of 24 time constants. Signal acquisition was carried out with the automatic adaptive tuning of bandwidth and input voltage range to achieve optimal measurement accuracy and sensitivity with the instrument.

Example of impedance data validation

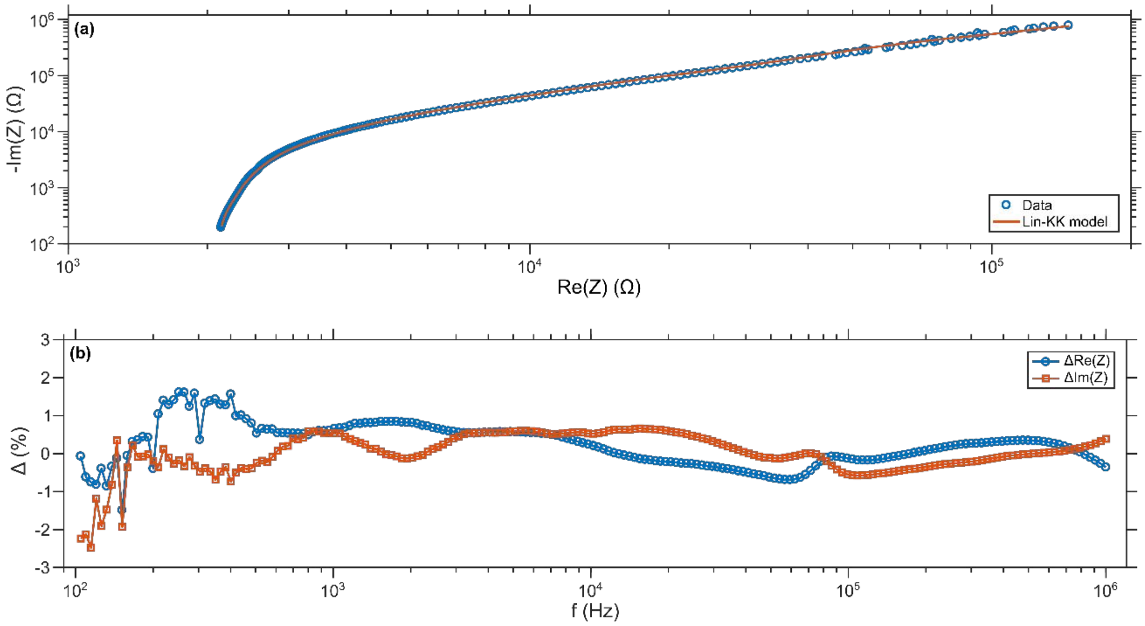

Figure S1. Validation of impedance data using the fitting with lin-KK model: (a) Nyquist plot indicating the good lin-KK model fit quality and (b) plot of lin-KK model fit residuals.

PEG layer formation

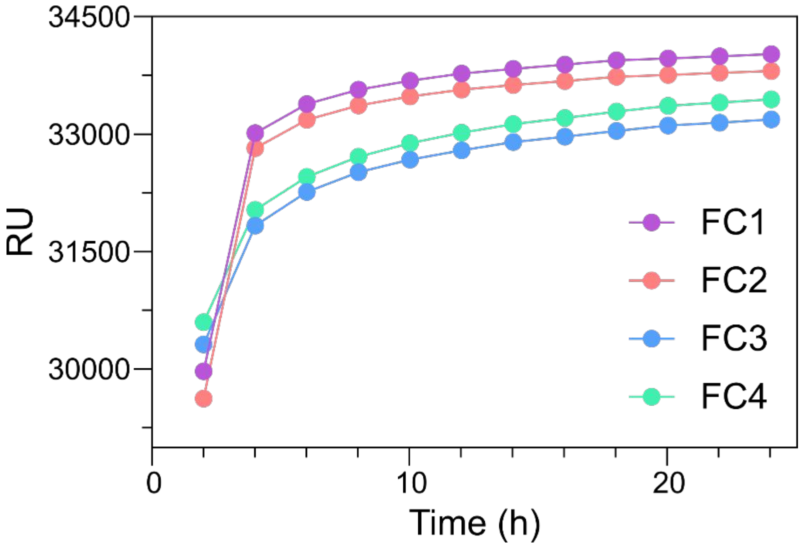

Figure S2. Formation of self-assembled monolayer (HS-PEG$_{5k}$-COOH) monitored by Surface Plasmon Resonance in four flow cells (FC1-FC4). After 24 h of incubation with HS-PEG$_{5k}$-COOH, the response stabilizes, indicating that the formation of the monolayer is complete.

Noise profile analysis for impedance sensing

Detailed analysis of the sensor noise profile was performed at different stages during the representative experiment of anti-SARS-CoV-2 IgG antibody (S1 Ab) detection in phosphate-buffered saline containing 0.1% Tween 20 (PBS-T) at ambient temperature. Noise profile of the sensor shows strong frequency dependence and remains relatively stable during different measurements (**Figure S3**). The noise spectrum at lower frequencies below 1 kHz is dominated by the contribution of power supply coupling while the imperfections in the measurement system connections have a dominant contribution at higher frequencies between 400 kHz and 1 MHz. Broad noise amplitude peak between 1 kHz and 300 kHz corresponds to the capacitive noise coupling between the sensor electrodes and the measurement environment. Analysis of the signal-to-noise ratio (SNR) (**Figure S4**) indicates higher reliability of the measurements performed at the frequencies above 1 kHz where the SNR values are typically above 70 dB. However, the sensor shows good SNR performance in the entire frequency range with all SNR values above 37 dB and the average SNR value of around 69 dB for the entire frequency range.

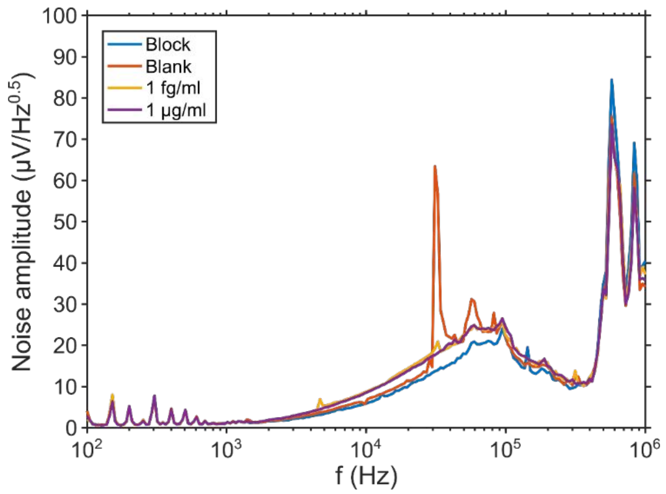

Figure S3. Noise profile of the impedimetric nanobiosensor in the frequency domain recorded at different stages of S1 Ab analyte detection in PBS-T medium at ambient temperature.

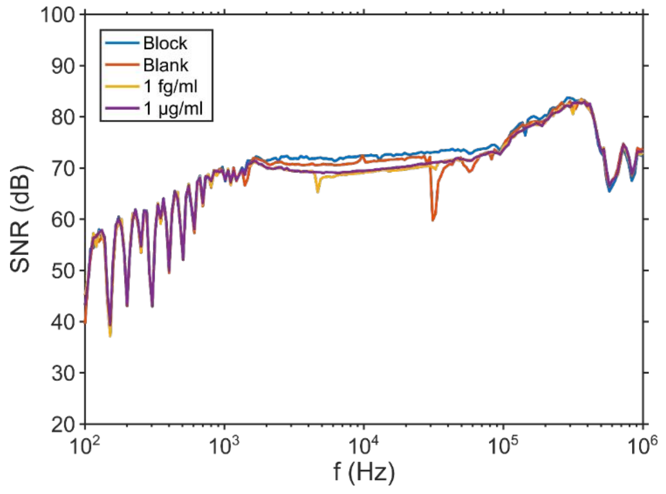

Figure S4. Frequency dependence of SNR calculated using the amplitude of the voltage signal recorded at the lock-in amplifier input during the acquisition of impedance spectra. Impedance spectra were acquired at different stages of S1 Ab analyte detection in PBS-T medium at ambient temperature.

Example of impedance data fitting

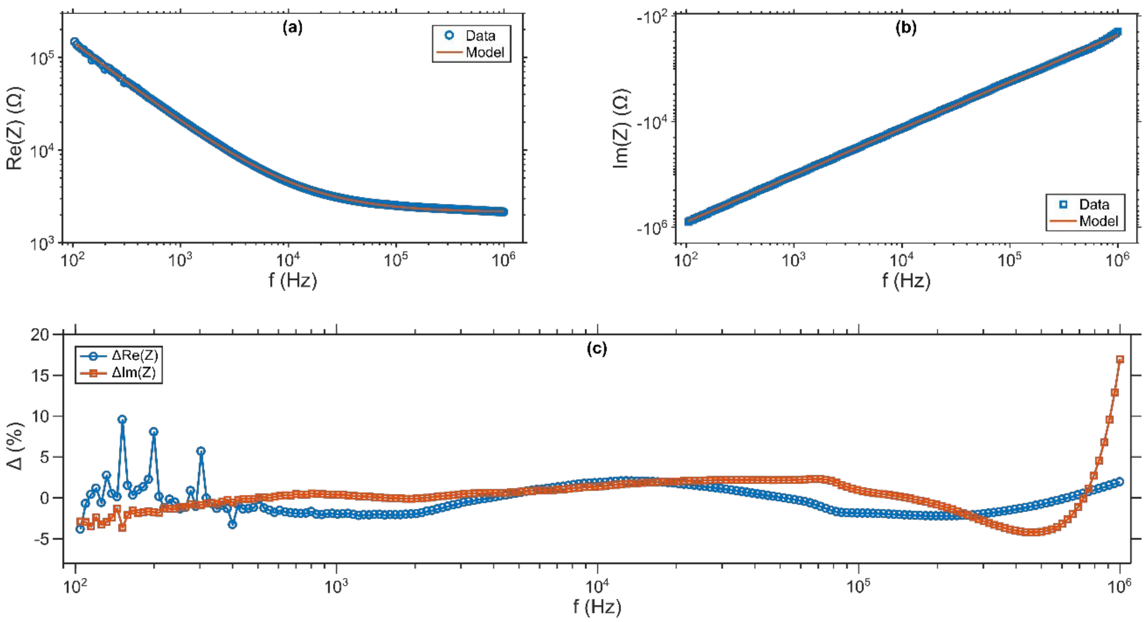

Figure S5. Example of impedance data fitting using our custom model: (a) fit for the real part of impedance data, (b) fit for the imaginary part of impedance data, and (c) plot of the custom model fit residuals.

Concentration of antibodies in clinical samples

Table S1. Concentration of antibodies determined by ELISA and sample classification of the samples obtained from COVID-19 recovered patients and unexposed patients.

Sample	Concentration of antibodies (µg/mL)	Classification (Control, moderate or high)
**P1**	12.22	High
**P2**	3.73	Moderate
**P3**	6.27	Moderate
**P4**	0.26	Control

Samples P1 to P3, which correspond to COVID-19 recovered patients, show moderate and high levels of anti-SARS-CoV-2 monoclonal antibodies. Conversely, sample P4 corresponds to a subject that was not infected with SARS-CoV-2, and thus is identified by this ELISA as control.

Experiments with clinical samples

The data used for the estimation of the concentration of the clinical samples using the nanobiosensor was normalized according to the following equation:

$$\Delta Z = \frac{|Z_a(1\ kHz) - Z_b(1\ kHz)|}{|Z_{blank}(1\ kHz) - Z_b(1\ kHz)|} \tag{S.5}$$

where is Z_a(1 kHz) the measured value of impedance modulus for a given concentration of the detected analyte at 1 kHz, Z_b(1 kHz) is the measured value of impedance modulus at 1 kHz in the blocking state, and Z_{blank}(1 kHz) is the measured value of impedance modulus at 1 kHz in the blank state (negative control).

The error bars (relative standard deviation) were calculated as follows:

$$Error = \Delta Z \sqrt{\left(\frac{\sqrt{{E_a}^2 + {E_b}^2}}{|Z_a(1\ kHz) - Z_b(1\ kHz)|}\right)^2 + \left(\frac{\sqrt{{E_{blank}}^2 + {E_b}^2}}{|Z_{blank}(1\ kHz) - Z_b(1\ kHz)|}\right)^2} \tag{S.6}$$

where E_a is the standard deviation of Z_a(1 kHz), E_b is the standard deviation of Z_b(1 kHz), and E_{blank} is the standard deviation of Z_{blank}(1 kHz).

Data analysis was performed with GraphPad Prism 9 (GraphPad Software, San Diego, CA, USA), and the calibration curve was fitted using the semilog line equation. To estimate the concentrations from the nanobiosensor response, the normalized data for the test well was interpolated using the normalized data from the calibration well.

Customized reliability levels were estimated as follows:

$$Reliability = 2x\frac{Error_1 + \ldots + Error_n}{N} \tag{S.7}$$

where $Error_1$ is the Error from the first concentration point (as calculated from Equation S.6), and $Error_n$ is the Error from the last concentration point (as calculated from Equation S.6), and N is the number of concentration points tested.

Calibration curves for experiments with clinical samples

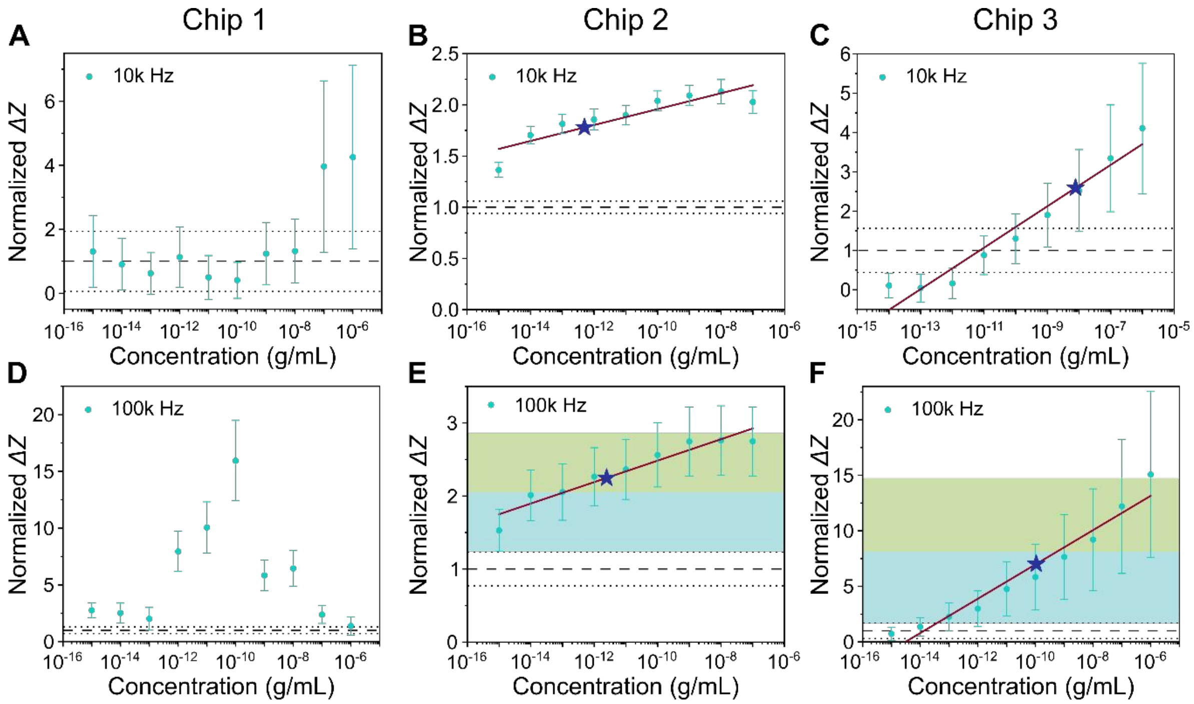

Figure S6. Calibration curves at 10 kHz for Chip 1 (A), Chip 2 (B), and Chip 3 (C). Calibration curves at 100 kHz for Chip 1 (D), Chip 2 (E), and Chip 3 (F). Values were normalized with respect to the blank. Dashed and dotted lines show the value of the blank and the relative standard deviation (see section **Experiments with clinical samples** in the **Supporting Information**), respectively. The blue star represents the estimated value of the concentration of S1 Abs of the subject tested in each chip (P2 in Chip 2 and P3 in Chip 3), calculated from the fitted line (red). Colored regions in (E) and (F) represent the reliability levels for the determination of the presence of S1 Abs in human samples, where green indicates higher reliability than blue (see section **Experiments with clinical samples** in the **Supporting Information**).

Sensor response for experiments with clinical samples

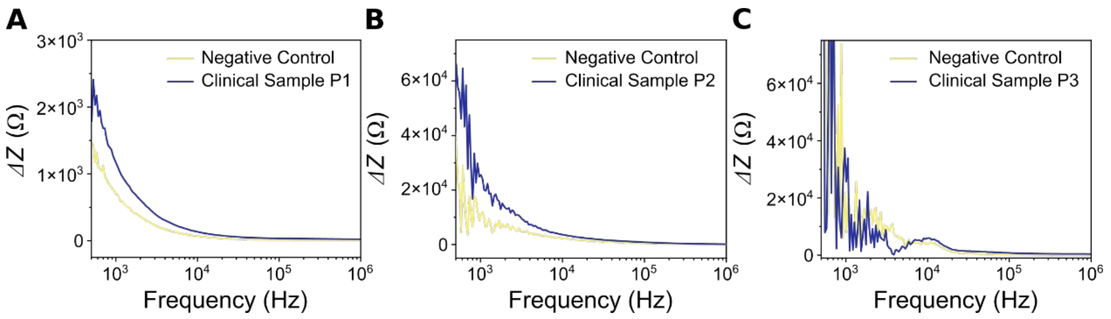

Figure S7. Signal response comparison of a seronegative COVID-19 subject (negative control) and three seropositive COVID-19 subjects: samples P1 (A), P2 (B), and P3 (C).

References

(1) Murbach, M. D.; Gerwe, B.; Dawson-Elli, N.; Tsui, L. Impedance.Py: A Python Package for Electrochemical Impedance Analysis. *Journal of Open Source Software* **2020**, *5* (52), 2349. https://doi.org/10.21105/JOSS.02349.

(2) Boukamp, B. A. A Linear Kronig-Kramers Transform Test for Immittance Data Validation. *Journal of The Electrochemical Society* **1995**, *142* (6), 1885. https://doi.org/10.1149/1.2044210.

(3) Schönleber, M.; Klotz, D.; Ivers-Tiffée, E. A Method for Improving the Robustness of Linear Kramers-Kronig Validity Tests. *Electrochimica Acta* **2014**, *131*, 20–27. https://doi.org/10.1016/J.ELECTACTA.2014.01.034.

(4) McKubre, M. C. H.; Macdonald, D. D. Measuring Techniques and Data Analysis. In *Impedance Spectroscopy: Theory, Experiment, and Applications*; Barsoukov, E., Macdonald, J. R., Eds.; John Wiley & Sons, Ltd, 2005; pp 129–204. https://doi.org/10.1002/0471716243.ch3.
